# Oral History in der Medizin – Herausforderungen und Chancen

**DOI:** 10.1007/s00048-024-00376-3

**Published:** 2024-02-27

**Authors:** Felicitas Söhner, Nils Hansson, Thorsten Halling

**Affiliations:** Institut für Geschichte, Theorie und Ethik der Medizin, Centre for Health and Society, Medizinische Fakultät, Heinrich-Heine-Universität Düsseldorf, Moorenstr. 5, 40225 Düsseldorf, Deutschland

## Einführung: Oral History als zeithistorisches Instrument

Die Oral History hat sich im Methodenspektrum der zeit- und wissenschaftshistorischen Forschung trotz wiederkehrender Skepsis inzwischen fest etabliert (Leh 2022). Viele neuere Interviewprojekte orientieren sich an aktuellen methodischen Standards und nutzen die verschiedenen Formen der medialen Darstellung. Bei genauerer Betrachtung wird deutlich, dass diese Annäherung vor dem Hintergrund unterschiedlicher Wissensbestände und oft aus unterschiedlichen Fachkulturen heraus erfolgt. Wir beobachten, dass die Fragen, die sich aus der wissenschaftlichen Arbeit mit der Oral History im deutschsprachigen Raum als Quellengattung, Forschungsmethode und interdisziplinäres Forschungsfeld ergeben, nur selten eingehend bedacht werden.

In diesem Forum möchten wir methodische Herausforderungen und ethische Fragen zur Oral History in der Medizingeschichte in Deutschland vor dem Hintergrund unserer Projekterfahrungen reflektieren und damit zu einer kritischen Reflexion der Potenziale der Oral History für die Medizin‑, Technik- und Wissenschaftsgeschichte einladen.

Reflektierte qualitative Interviewprojekte hielten im internationalen Vergleich (Tomes 1991; Bornat 2000) in der deutschsprachigen Medizingeschichte erst relativ spät Einzug (Clark 2005; Atzl et al. 2005; Kreutzer 2006; Herrn & Hottenrott 2010). Insbesondere jüngere zeithistorische Forschungsprojekte zum Themenkomplex von Unrechtserfahrungen und Missbrauch in Institutionen, die dem Schutz vulnerabler Personen verpflichtet waren, steigerten das Problembewusstsein für methodische und ethische Herausforderungen einer anspruchsvollen historischen Methode, die weit über eine Ansammlung von subjektiven Erinnerungen und Anekdoten hinausgeht (Arp 2017; Söhner 2017).

Projekte mit biografischen Interviews müssen daher differenziert betrachtet werden: In der fachlichen Debatte wird der Unterschied zwischen Zeitzeug*innen- und Oral-History-Interviews diskutiert und auf die Herausforderung verwiesen, gegenüber Zeitzeug*innen als mündlicher Quelle – wie gegenüber allen historischen Quellen – eine kritisch reflektierte Haltung einzunehmen (Thomson 1988; Wierling 2014). Um diesen Anspruch zu erfüllen, werden in der Analyse und Interpretation von Oral-History-Gesprächen – im Vergleich zu einfachen Interviewprojekten – beispielsweise nicht nur der Wortlaut, sondern auch die Auslassungen, Brüche und Widersprüche berücksichtigt, die gegebenenfalls mehr über die Wahrnehmung und Darstellung der Befragten aussagen als das eigentlich Erzählte (Portelli 1991: 53).

Die geschichtswissenschaftliche Forschung interessiert sich in erster Linie für die erzählte Biografie als Trägerin und Verarbeitung historischen Wissens und historischer Erfahrung. Im methodischen Kanon der Sozialwissenschaften nehmen biografische Ansätze einen festen Platz ein (Lutz et al. 2018). In soziologischen Zugängen wird die Tendenz einer ahistorischen Sichtweise als Schwäche kritisiert sowie die Illusion, man könne gegenwärtige Fragen ohne Bezug auf die Vergangenheit klären. An der historischen Rekonstruktion hingegen wird die Neigung zur Vernachlässigung der Gegenwartsperspektive bemängelt und daher eine gezielte, methodisch kontrollierte Rekonstruktion erlebter Vergangenheit und eine Einbettung in die tradierte Kollektivgeschichte vorgeschlagen (Rosenthal 2015).

Die Oral History nähert sich insbesondere Fragestellungen, zu denen kaum schriftliche Quellen vorliegen oder diese aufgrund archivalischer Schutzfristen noch nicht zugänglich sind, wie auch subjektiven Erfahrungs- und Verarbeitungsprozessen historischer Ereignisse und Prozesse (Te Heesen 2021). Über den mündlichen Zugang lassen sich subjektive Wahrnehmungs- und Interpretationsmuster vergangener Ereignisse und Prozesse untersuchen (Wierling 2003). Ihr Hauptwert liegt also in der Aufzeichnung von Wahrnehmungen und Erinnerungen – nicht in der Ermittlung spezifischer Fakten oder Ereignisse. Zentrale Herausforderung der Oral History ist, dass die Forschenden ihre Quellen selbst erstellen und damit auch für deren Qualität verantwortlich sind. Daher muss die Gesprächsführung und Dokumentation festgelegten Gütekriterien entsprechen.

In der Medizingeschichte können beispielsweise Perspektiven marginalisierter Personen- und Berufsgruppen im Gesundheitswesen auf sich verändernde medizinische Behandlungsweisen und -strukturen beleuchtet werden (Hansson et al. 2022). Dabei lassen sich hegemoniale Narrative identifizieren, die zu einer Neubewertung historischer Ereignisse und Prozesse beitragen (Niethammer 1985; Hoyle 2022). Im Idealfall geben biografische Interviews auch Aufschluss über das Wechselverhältnis zwischen Strukturen in der Medizin und individuellen Erfahrungen und ermöglichen es, intellektuelle Prozesse der Generierung von Wissen nachzuvollziehen (Fisher 2011; Vargha 2018). Im Sinne einer kritischen Standortbestimmung für Forschende in der Wissenschaftsgeschichte, die den speziellen Status der Oral History in der Medizin verstehen und reflektieren möchten, werden methodische und praxeologische Herausforderungen von Forschungsprojekten im deutschsprachigen Raum im Hinblick auf die Frage ausgeleuchtet, ob und inwiefern Oral-History-Studien in der Wissenschafts-, insbesondere aber in der Medizingeschichte spezifischen methodischen Anforderungen unterliegen.

## Methodische und praxeologische Überlegungen

Forschungsethische und forschungspraktische Herausforderungen von Oral-History-Projekten in der Medizingeschichte gestalten sich je nach den Bedürfnissen der involvierten Personen unterschiedlich. Aufgezeigt wird dies anhand der für die Oral History grundlegenden Forschungsschritte. Diese beinhalten die forschungsethischen Fragestellungen, die Herstellung eines Vertrauensverhältnisses, die Erhebung und die Auswertung von Daten, die Darstellung der Ergebnisse sowie die Reflexion des Forschungsprozesses (Köppen et al. 2020).

### Oral History und Forschungsethik

Medizinhistorische Forschungsprojekte, die mithilfe von Interviews und Fragebögen Daten von Befragten erheben, um etwa Bedingungsfaktoren von Gesundheit und Krankheit zu erforschen, können unter ethischen Aspekten auch der sogenannten „Forschung am Menschen“ zugerechnet werden (Roelcke 2007).

An diese Studien werden in der Medizin besonders hohe Anforderungen an Transparenz und Dokumentation gestellt. So ist für Oral-History-Projekte, die an medizinischen Fakultäten in Deutschland durchgeführt werden, vor Beginn des Forschungsprojekts das Vorlegen eines Antrags vor der Ethikkommission der jeweiligen medizinischen Fakultät erforderlich. Dies bedeutet für die beteiligten Forschenden, dass die im Kontext experimenteller oder klinischer Forschung etablierten Begrifflichkeiten und Kategorien auf medizinhistorische Forschungsprojekte übertragen und angewendet werden müssen. Die Voraussetzung eines positiven „Ethikvotums“ setzt sich im deutschsprachigen Raum zunehmend disziplinübergreifend durch – so auch für die mündliche Geschichtsschreibung (wie PSH Berlin 2014, JGU Mainz 2015, U Passau 2019, TU Chemnitz 2021).

Medizinische Forschungsprojekte sollten im Vorfeld ihrer Durchführung daraufhin überprüft werden, ob sie „das Leben, die Gesundheit, die Würde, die Integrität, das Selbstbestimmungsrecht, die Privatsphäre und die Vertraulichkeit persönlicher Informationen“ der beteiligten Personen schützen (Art. 9, Deklaration von Helsinki). Als Ausdruck des Selbstbestimmungsrechts gilt eine „informierte Einwilligung“ als unabdingbare Voraussetzung jeglicher Forschungsprojekte. Sie basiert auf drei Bedingungen: der Einwilligungsfähigkeit beziehungsweise einer Entscheidungskompetenz, einer verständlichen Aufklärung sowie der Freiwilligkeit der Entscheidung. Hier gilt es, Faktoren zu minimieren, die eine freiwillige Entscheidung der Teilnehmenden beeinflussen können – wie etwa moralischen Druck, finanzielle Anreize, Hierarchiegefälle. Zentrales Kriterium bei der ethischen Beurteilung von Forschungsprojekten ist die Risiko-Nutzen-Abwägung: Rechtfertigt der erwartete Erkenntnisgewinn der Studie die möglichen Komplikationen? Lassen sich Erkenntnisse möglicherweise ebenso ohne Interviews erlangen? Solche Fragen stellen sich insbesondere dann, wenn traumatisierende Erinnerungen erfragt und damit möglicherweise reaktiviert werden.

Wie in der Shoah-Forschung ist es auch hier forschungsethisch geboten, Belastungen der Betroffenen zu antizipieren und mögliche Unterstützungsressourcen zur Verfügung zu stellen (Spitta 2009). Dies ist im Besonderen erforderlich, wenn vulnerable Personen befragt werden. Gemeint sind Personen, für die eine besondere körperliche oder seelische Verletzlichkeit und daher eine erhöhte Schutzbedürftigkeit angenommen wird (Wild 2014). Mögliche Gründe für eine so verstandene Vulnerabilität können im Lebensalter oder aber auch in einer eingeschränkten Entscheidungsfähigkeit liegen, zum Beispiel aufgrund einer Demenz oder einer psychischen Erkrankung. Bei der forschungsethischen Beurteilung solcher Studien spielt eine wesentliche Rolle, ob deren Ergebnisse den involvierten Personen selbst nutzen. Im Kontext wissenschaftshistorischer Projekte könnte dies beispielsweise durch eine Gedächtnisstiftung oder als Beitrag zu einer gesellschaftlichen Anerkennung von erlebtem Leid der Fall sein.

Auch Fragen zum Schutz personenbezogener Daten, der Vertraulichkeit von Gesprächsinhalten sowie der Einhaltung rechtlicher Maßgaben zum Schutz individueller Rechte haben eine hohe Relevanz. In Projekten, in denen professionell Involvierte befragt werden, geht es vor allem um den Schutz Dritter, die in den Gesprächen erwähnt werden.

### Erhebung von Daten

Eine große Herausforderung bereits in der frühen Projektphase ist die Berücksichtigung der Datensicherheit: Die Speicherung audiovisueller Dokumente wie der dazugehörigen Aufzeichnungen bei gleichzeitiger Sicherheit sensibler medizinischer Daten bedeuten für die beteiligten Institute und Archive technische Herausforderungen. Neue Zielkonflikte bahnen sich aktuell durch die Anforderungen an ein transparentes Forschungsdatenmanagement im Rahmen von Drittmittel-geförderten Projekten (Steinhardt et al. 2020) an.

In Oral-History-Projekten – auch in der Medizin – treten Fragen auf zur Zusammensetzung und Sättigung der Stichprobe. Theoretische Sättigung wird dahingehend verstanden, dass im Datenmaterial durch die Hinzufügung weiterer Aussagen kaum neue, relevante Informationen ergänzt werden und somit verallgemeinerbare Aussagen gerechtfertigt erscheinen (Fuchs-Heinritz 2000). Dieser Aspekt lässt sich nicht einfach interdisziplinär übertragen. Hierzu ist noch eine spezifische methodische Debatte angezeigt.

Wenn die Entscheidung getroffen wurde, die Fragestellung über qualitative Interviews (narrativ, leitfadengestützt) zu beantworten, muss die Datenerhebung an die involvierten Personen und deren Kompetenzen und Bedürfnisse angepasst werden. Vor der eigentlichen Erhebung sind nochmals Ziele und Ablauf der Erhebung zu klären; auch ist ein Methodentraining für involvierte Forschende sinnvoll.

Der Ablauf der Interviews wie die Gesprächsinhalte werden nicht nur durch die Wahl der Befragten und explizite Impulse, sondern auch durch das para- und nonverbale Verhalten der Interviewenden beeinflusst. Befragte verhalten sich bewusst wie unbewusst zur professionellen Herkunft der Fragenden. So zeigt sich, dass Beruf und Habitus der Interviewenden das Gespräch bereits rahmen, bevor ein erstes Wort gesprochen wird (Wierling 2003).

In der Analyse sollte der Aspekt der Teilnahmemotivation berücksichtigt werden. Diese kann neben der Selbstdarstellung von Person oder Institution auch die Korrektur von historischen Narrativen, die Rechtfertigung früheren Verhaltens oder auch die Repräsentation einer Gruppenidentität sein (Baehrens 2013).

Weiter gilt es zu beachten, dass die Befragten nicht nur als erfahren im erfragten Themenfeld, sondern auch als Angehörige einer professionellen oder generationellen Perspektive verstanden werden können.

Für Oral-History-Projekte in der Medizingeschichte bedeutet dies, bereits vor der Gesprächsführung historische professionelle Entwicklungen und fachpolitische Diskurse zu kennen, um individuelle Reaktionen im Interview verstehen und ihnen angemessen begegnen zu können. Im Vergleich zur Oral History im Allgemeinen kann dies einen höheren Aufwand bei der thematischen Einarbeitung in das jeweilige Themenfeld bedeuten.

### Herstellung eines Vertrauensverhältnisses

Aufgrund des lebensgeschichtlichen Bezugs treten die Forschenden in ein Vertrauensverhältnis zu den Interviewten; daher sind damit einhergehende Fragen und Verpflichtungen zu reflektieren.

Ein wichtiges Element der biografiegestützten Forschung und zugleich eine Voraussetzung für deren erfolgreiche Umsetzung ist ein geschützter Kommunikationsraum, der von Offenheit und Vertrauen geprägt ist (Wicks & Reason 2009). Die Ansprüche an diesen Kommunikationsraum, meistens beginnend mit der Interviewsituation (u. a. persönlich vs. online, im Lebensumfeld der Gesprächspartner vs. frei gewählte oder vom Interviewenden vorgegebe Gesprächsorte), müssen an die Erwartungen und Fähigkeiten der Interviewten angepasst werden. Nicht immer, aber doch häufig gehören beispielsweise die Wahrung der Persönlichkeitsrechte, gegebenenfalls die Einbindung von Vertrauenspersonen wie auch die Herstellung kommunikativer Offenheit zu diesem Prozess. Individuelle Schicksale müssen im Rahmen von Publikationen gegebenenfalls so verfremdet werden, dass ein Wiedererkennen noch lebender Interviewter verhindert wird. Um gleichzeitig die Prinzipien guter wissenschaftlicher Praxis wie Transparenz und Nachprüfbarkeit der Quellen zu gewährleisten, sollten entsprechende Vorgehensweisen mit Peer-Forschenden abgestimmt und Interessen offengelegt werden. Bei der Anpassung der Methoden an die Bedürfnisse vulnerabler Personen können die von der ICPHR 2013 formulierten spezifischen Kriterien zu partizipativer Gesundheitsforschung ein guter Anhaltspunkt sein.

Die Wahrung von Privatsphäre und Vertraulichkeit im Zusammenhang mit sensiblen Themen in Oral-History-Projekten, deren Ziel im Besonderen das Erfragen von individuellen Erinnerungen und Lebensschicksalen ist, stellt besonders hohe Herausforderungen an den Datenschutz. Datenschutzkonzepte sollten nicht nur Aspekte der Datenverarbeitung (z. B. Pseudonymisierung vs. Anonymisierung) beinhalten, sondern auch Fragen zur Archivierung beantworten. Die nach Abschluss medizinischer Studien in aller Regel durchgeführte Vernichtung von Daten nach einem bestimmten Zeitintervall – etwa nach zehn Jahren – läuft dabei dem historischen Interesse an einer Bewahrung von historischen Forschungsquellen (so auch biografische Interviews) zuwider.

In den verschieden verstandenen Notwendigkeiten von Transparenz, Datenschutz und Archivierung liegt daher eine besondere Herausforderung für medizinhistorische Interviewprojekte. Die Frage des Doppelstatus einer Aufzeichnung als Studienmaterial beziehungsweise klinische Dokumentation und historische Quelle findet sich auch bei schriftlichen Aufzeichnungen wieder (vgl. historische Krankenakten) und bedarf eines alle Interessen berücksichtigenden rechtssicheren Vorgehens.

### Auswertung von Daten

Auch in der Auswertung der Daten sind in medizinhistorischen Projekten spezifische Aspekte zu berücksichtigen. Individuelle Erinnerungen lassen sich als Ergebnis komplexer kommunikativer Prozesse verstehen, die durch soziale, institutionelle und individuelle Deutungsmuster wie auch von latenten wie manifesten Erwartungen überformt sein können. Gleichzeitig lassen sich individuelle Identitätskonstruktionen basierend auf dem menschlichen Grundbedürfnis nach Anerkennung und Zugehörigkeit einordnen.

Autobiografische Lebenslaufkonstruktionen sind überwiegend selbstrepräsentierend (Einzelner, Gruppe, Institution, Profession) und scheinen kaum „*identitätsneutral*“ zu sein (Reinhard 2006). Die formulierten Erinnerungen lassen sich als Indikator der Persönlichkeit der Befragten und ihres privaten wie professionellen Identitätsgefühls verstehen (Söhner 2020). Damit sagen biografische Erinnerungen vor allem etwas darüber aus, wie zurückliegende Ereignisse und Prozesse wahrgenommen und beurteilt, wie diese derzeit erinnert und auch seitens der Befragten präsentiert werden wollen.

In Projekten mit professionellen Akteur*innen im Gesundheitswesen sind zumeist Aspekte bedeutsam wie das ärztliche Vertrauensverhältnis und fachkulturelle Prägungen, die mit Leistungsgedanken und Statusdenken einhergehen können. Interessant ist in diesem Zusammenhang die Frage der Selbsteinschätzung und Selbstdarstellung professioneller Personen in der Medizin, insbesondere vor dem Hintergrund der dargestellten Professionsgeschichte (Dörner 2001). Nicht unerheblich erscheinen uns in diesem Zusammenhang auch Verschiebungen bei gesellschaftlichen Moralvorstellungen, aber auch bei medizinethischen Fragen (Unschuld 2009). Hier gilt es zu reflektieren, inwiefern sich dies auf Erinnerungen an eigene Handlungen auswirkt.

Auch die Frage des Zeitgeists spielt in der Analyse aller Oral-History-Dokumente eine Rolle. In Projekten der Medizin- oder Wissenschaftsgeschichte bedeutet dies, den gesellschafts- und gesundheitspolitischen Diskurs zum untersuchten Feld im Erhebungs- und im Auswertungszeitraum in die Analyse einzubeziehen. Auch die den Akteur*innen im untersuchten Feld zugeschriebenen Rollen wie auch internalisierte Selbstverständnisse sind in besonderem Maße zu beachten.

### Darstellung der Ergebnisse

Mit der Analyse einher geht die Frage der Darstellung der erarbeiteten Ergebnisse. Der methodische Ansatz der Oral History möchte einen direkten Zugang zu den Perspektiven der Beteiligten eröffnen, der über die überlieferten Informationen aus Akten und anderweitigen Dokumenten hinausgeht. Damit möchte sich auch die medizinhistorische Forschung die Möglichkeit verschaffen, mündliche Überlieferungen in Form von individuellen Meinungen, Einstellungen, Ereignissen oder Erfahrungen zu erheben und zu analysieren (Erll 2017). Über die Rekonstruktion von Lebenswirklichkeiten aus professioneller Perspektive lassen sich nicht nur Alltags- und Machtstrukturen, sondern auch Fragen der Verantwortung im untersuchten Feld erarbeiten.

In der Darstellung der Ergebnisse sind persönliche Motivationen der beteiligten Personen zu bedenken. Beispielsweise in den Projekten mit vulnerablen Personen, zu „Leid und Unrecht“ (Fangerau et al. 2021) und „Testimony“ (Söhner et al. 2021) boten neben medialen und Fachbeiträgen auch Betroffenenveranstaltungen den Beteiligten die Möglichkeit, sich in einem fachlich begleiteten Rahmen zu informieren, auszutauschen und Anliegen Ausdruck zu verleihen. In elitenerforschenden Projekten wie „Bridging the Baltic“ (Hansson 2022; Hansson et al. 2022) bietet neben mehrsprachigen Publikationen und Präsentationen eine Internetplattform den beteiligten Expert*innen die Möglichkeit, ihre Erfahrungen in öffentlich zugänglichen Interviews zu teilen.

### Reflexion des Prozesses

Während der eigentlichen Arbeitsschritte ist die Reflexion des Forschungsprozesses eine Aufgabe, die Projekte durch alle Phasen begleiten sollte.

Bei Oral-History-Projekten mit vulnerablen Personen sind Fragen der Selbstermächtigung, der Wiederaneignung von Geschichte und der Nachhaltigkeit des Projekts zu prüfen und damit einhergehend die Frage, inwiefern sich Konsequenzen für die Praxis ableiten lassen.

Bei Projekten mit Angehörigen professioneller Eliten in der Medizin werden die Besonderheiten der Interviewführung nochmals besonders deutlich. Die befragten Personen sind es in der Regel gewohnt, selbstbewusst eine klare Position zu vertreten und zu wissen, welche Themen sie auf welche Weise rahmen und transportieren wollen. Die berufliche Sozialisierung der Expert*innen im Feld der Medizin beinhaltet eine starke innere Verpflichtung. Inhaltlich sind es daher nicht selten hegemoniale Narrative, die die Interviewten formulieren, wiedergeben oder auch einfordern können, für die sich die methodische Frage der Identifizierung und Deutung charakteristischer Erzählmuster beziehungsweise der Einordnung und Analyse verfestigter Formulierungen stellt (Söhner 2024).

Dies bedeutet für die Oral History in der Wissenschafts- und Medizingeschichte, dass ein erhöhtes Maß an selbstkritischem Vorgehen im Forschungsprozess nötig ist.

## Oral History in der Medizin – etwas Besonderes?

Eine zentrale Bedeutung der Oral History im Allgemeinen besteht darin, historische Ereignisse und Prozesse individualisieren zu können. Dabei lassen sich historische Begebenheiten nicht nur anschaulicher festhalten, sondern auch Perspektiven dokumentieren, die über schriftliche Quellen kaum zugänglich wären. Oral History analysiert die Konstruktion der Erinnerung; die Herausforderung liegt darin, das Erzählte von der Erfahrung zu unterscheiden (Broda 2004).

Aufgrund der gesellschaftspolitischen Wirkung wissenschaftlicher Wissensproduktion in Wissenschaft, Medizin und Technik erfordert der biografisch-historische Ansatz Sensibilität, da Erinnerung und Gedächtnis dynamisch funktionieren sowie zustands- und kontextabhängig sind. Vorherrschende Narrative betten individuelle Haltungen, Entscheidungen und letztendlich Handlungen diskursiv ein und beeinflussen sowohl die seinerzeitige Wahrnehmung von Ereignissen und Prozessen wie auch letztlich die Erinnerung daran. Wir verstehen es als eine daraus resultierende permanente Aufgabe, den subjektiven Charakter persönlicher Erinnerungen zu reflektieren und die Perspektive der sich Erinnernden historisch zu kontextualisieren (Berek 2009).

Über den Einblick in diachrone Perspektiven, Erfahrungen und Überzeugungen ermöglicht es die Oral History in der Medizin, große institutionelle Strukturen, die Rolle einflussreicher Persönlichkeiten, die „günstigen Gelegenheiten“ von Ort und Zeit und den Kontext kritischer Entscheidungen nachzuzeichnen und ein Stück weit zu entmystifizieren. Auch trägt dieser Zugang dazu bei, zurückliegende Motivationen, Erwartungen und Sorgen angesichts fachlicher Entwicklungen (Professionalisierung, Spezialisierung, Bürokratisierung, Digitalisierung) historisch nachzuvollziehen. So kann die mündliche Geschichtsschreibung des Faches entscheidend zu einem historischen Verständnis der Transformation der Medizin beitragen.

Vor diesem Hintergrund stellt sich nun die Frage, ob und inwiefern Oral History in der Medizin als etwas Besonderes verstanden werden kann.

Ein besonderer Aspekt ist, dass in der Medizingeschichte angesiedelte Oral-History-Projekte, die mit Menschen als Interviewten forschen, unter Umständen als „Forschung am Menschen“ verstanden werden können. Dies wirkt sich bereits auf praxeologische Überlegungen in der Konzeptionsphase von Projekten aus.

Zu den spezifischen methodisch-analytischen Herausforderungen für Oral-History-Projekte mit professionell Handelnden in der Medizin gehört die frühzeitige Klärung ethischer und rechtlicher Zielkonflikte im Spannungsfeld von historischem Erkenntnisinteresse und der Wahrung von Persönlichkeitsrechten der Beteiligten.

Zusammenfassend lässt sich festhalten, dass die hier angeführten speziellen, klinisch orientierten Besonderheiten historische Forschung beeinflussen und es bei der Oral History in der Medizin Unterschiede im Arbeitskontext gibt. Gerade weil diese in der Publikation der Ergebnisse nicht explizit sichtbar werden, sind sie in interprofessionellen Projekten abzustimmen, in der methodischen Darstellung in Publikationen zu reflektieren und im fachlichen Austausch innerhalb der Medizin- und Wissenschaftsgeschichte sowie der Oral History zu kommunizieren.

Oral-History-Interviews stellen für die Medizin- und Wissenschaftsgeschichte Quellen zur Verfügung, die es erlauben, bislang offene Fragen zu bearbeiten – insbesondere, wenn Archivbestände nicht oder noch nicht zugänglich sind. Weiter kann die Oral History dazu beitragen, Hierarchien aufzuweichen, indem Personen aus dem untersuchten Bereich selbst einen Beitrag zur Geschichtsschreibung leisten. Der Zugang erlaubt es, bisherige Narrative zur Medizin- oder Wissenschaftsgeschichte zu hinterfragen, indem vielfältige Perspektiven auf und aus dem Fach sichtbar werden und damit zusammenhängende Argumentationsmuster und deren Einbettung in den fachlichen oder gesellschaftlichen Diskurs eingeordnet werden können. Damit lassen sich individuelles und institutionelles Handeln sowie fachliche Entwicklungen nachvollziehen.
